# Tumor-to-Tumor Metastasis to Chromophobe Renal Cell Carcinoma: A First Report

**DOI:** 10.1155/2011/520839

**Published:** 2011-10-05

**Authors:** Toshitaka Shin, Tomoko Kan, Fuminori Sato, Hiromitsu Mimata

**Affiliations:** Department of Urology, Faculty of Medicine, Oita University, 1-1 Idaigaoka, Hasama-machi, Oita 879-5593, Japan

## Abstract

Tumor-to-tumor metastasis is a rare phenomenon. From our review of the international literature, around 150 cases have been reported since it was first documented by Campbel in 1868. Renal clear cell carcinoma is well known to be the most common recipient of tumor-to-tumor metastasis in all tumors. However, renal chromophobe cell carcinoma has not been reported to be a recipient. Here, we report a first case of colorectal carcinoma metastatic to chromophobe renal cell carcinoma.

## 1. Introduction

The occurrence of various cancers metastasizing to different recipient host tumors is extremely rare [1, 2]. Renal clear cell carcinoma is the most common recipient tumor of metastasis while chromophobe cell carcinoma has not been reported to be a recipient [3, 4]. We present a case of colorectal carcinoma metastatic to chromophobe renal cell carcinoma and a brief review of the relevant literature.

## 2. Case Report

**Figure 1:**
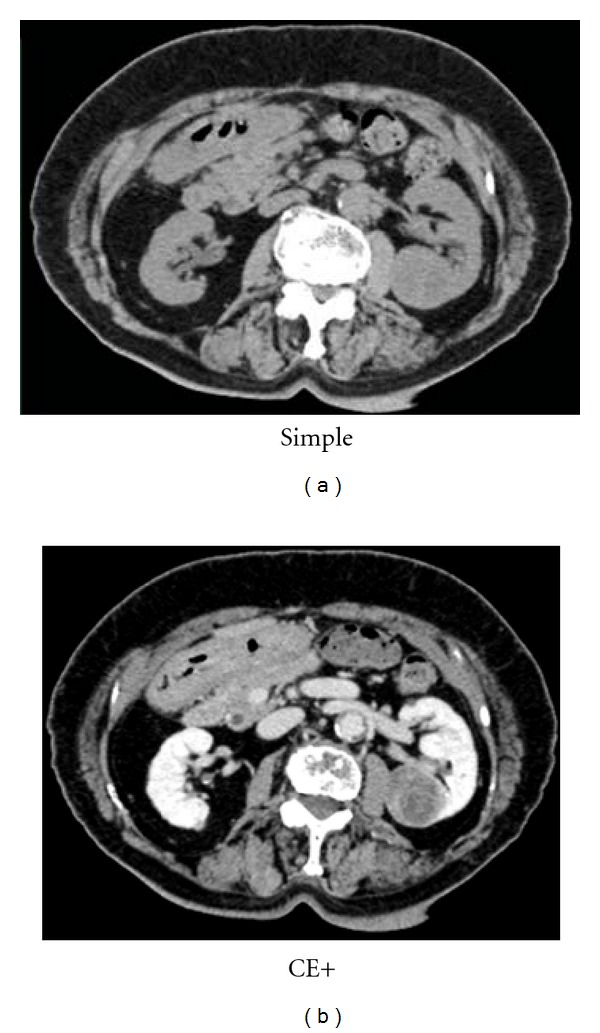
Computed tomography at the time of diagnosis. It showed a heterogeneously enhanced left renal mass 35 mm in diameter.

**Figure 2:**
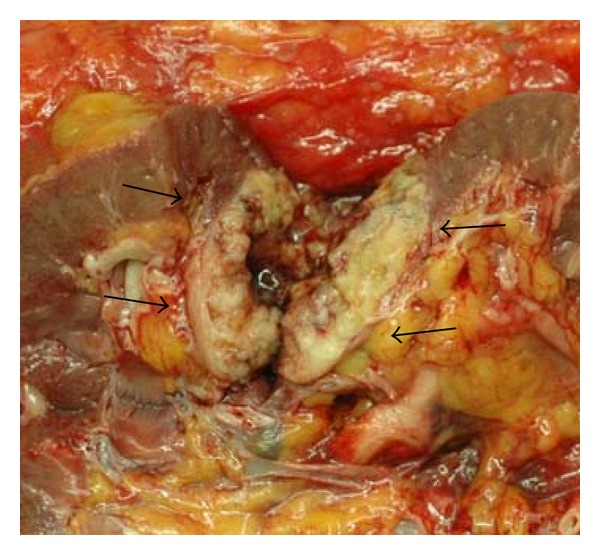
A gross pathologic specimen. Sectioning revealed a light yellow well-circumscribed mass in the middle portion of the kidney, measuring 3.5 × 3.8 cm. The tumor contained the hemorrhage or necrosis at inside.

**Figure 3:**
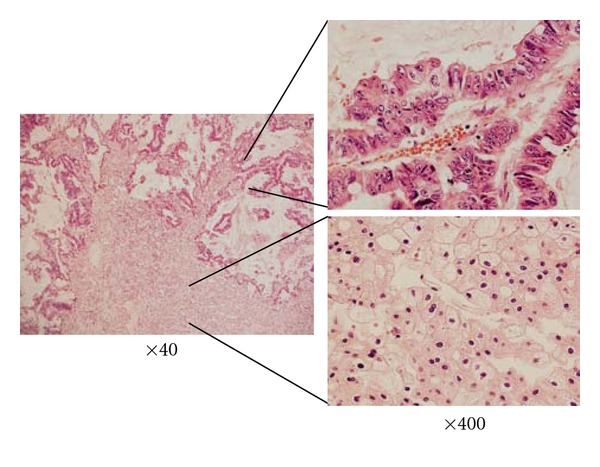
Histologic appearance of junction between metastatic poorly differentiated adenocarcinoma (upper) and chromophobe renal cell carcinoma (lower), HE staining.

A 82-year-old woman was referred to our hospital for evaluation and treatment of the left renal mass. She has a history of simultaneous ascending colon and rectal adenocarcinoma, which were both resected and fecal diversion was placed. Single liver metastasis was also resected at the same time. Next year, left lobectomy was performed to resect a new single lung metastasis. After 2 years from the first operation, computed tomography (CT) scan showed an enlarging heterogenous left renal mass 35 mm in diameter (Figure 1). In contrast enhancement (CE) CT, the tumor was gradually enhanced, at peripheral lesion in particular. Therefore, this tumor was thought likely to be a primary renal cell carcinoma or metastatic carcinoma. Retroperitoneoscopic left nephrectomy was performed. Sectioning revealed a light yellow well-circumscribed mass in the middle portion of the kidney, measuring 3.5 × 3.8 cm (Figure 2). The tumor contained the hemorrage or necrosis at inside. Microscopically, the tumor was composed largely of two patterns of small oxyphilic granular cells and larger light translucent cells when using the hematoxylin and eosin (HE) staining (Figure 3 upper). These features are consistent with chromophobe cell carcinoma of the kidney. However, within the chromophobe renal cell carcinoma, there were another foci of atypical cells arranged in high-columnar pattern (Figure 3 lower). These cells grew with the production of viscous liquid and the necrosis. These findings were as same as those of the previous colorectal adenocarcinoma. Thus, pathological diagnosis was colorectal carcinoma metastatic to chromophobe renal cell carcinoma. Then, the patient was merely put under followup and is still without evidence of disease, 8 months after the nephrectomy.

## 3. Discussion

Although the coexistence of 2 or more primary neoplasms in the same patient is sometimes observed, tumor-to-tumor metastasis is a rare phenomenon [1–4]. Documentation of tumor-to-tumor metastasis must meet certain criteria. According to Campbell et al. [1] these criteria are the following [2, 3]: (1) more than 1 primary tumor must exist; (2) the recipient tumor is a true benign or malignant neoplasm; (3) the metastatic neoplasm is a true metastasis with established growth in the host tumor, not the result of contiguous growth (collision tumor) or embolization of tumor cells; (4) tumors that have metastasized to the lymphatic system, where lymphoreticular malignant tumors already exist, are excluded. 

Many of the cases reported previously were discovered only at autopsy. In this phenomenon, the most frequent recipient is clear cell carcinoma of the kidney, followed by sarcomas, meningiomas, and thyroid neoplasms, while the most common donor is carcinoma of the lung, followed by carcinoma of the breast, gastrointestinal tract, prostate, and thyroid [2]. Two factors may contribute to the preferential homing of metastatic cancer to renal clear cell carcinoma [2, 3, 5, 6]. One is the rich vascularization of clear cell carcinomas, which renders them more accessible to metastatic tumor cells in the circulating blood. This theory is called “*mechanical theory.*” The other is the high lipid and glycogen content in clear cell carcinoma, which may provide a nutrient-rich microenvironment for metastatic tumor cells. This theory is called “*seed and soil theory.*” 

However, chromophobe cell carcinoma does not have these special features and other unknown mechanisms might work for this phenomenon. Chromophobe renal cell carcinoma is an uncommon variant of renal cell carcinoma, accounting for approximately 3–5% of renal cancer [7]. Chromophobe cell carcinoma has distinct biologic and clinical characteristics compared with clear cell carcinoma. The typical cytological findings are a light translucent, but not empty cytoplasm when using the hematoxylin and eosin staining [7]. The cells are usually voluminous with pronounced cell boundaries. Clinically, chromophobe cell carcinoma is considered to have a good prognosis.

To our knowledge, this is the first reported case of tumor-to-tumor metastasis to chromophobe cell carcinoma. 

Although tumor-to-tumor metastasis occurs infrequently, this possibility should always be considered when an unusual dimorphic pattern appears in a tumor. The phenomenon may become more frequent because of the improving prognosis and survival of patients with malignancies.
